# Caffeine Intoxication in Pregnancy; a case Report

**Published:** 2019-11-16

**Authors:** Tsuyoshi Nojima, Hiromichi Naito, Yoshinori Kosaki, Takaaki Osako, Kimiaki Tanaka, Atsuo Murata, Atsunori Nakao

**Affiliations:** 1 Emergency Department, Critical Care and Disaster Medicine, Okayama University Graduate School of Medicine Dentistry and Pharmaceutical Sciences, Okayama, Japan.; 2 Department of Emergency Medicine, Kochi Health Science Center, Kochi, Japan.

**Keywords:** Caffeine, hemodiafiltration, pregnancy, poisoning

## Abstract

Although fatalities due to caffeine intoxication are uncommon, a caffeine overdose may cause profound toxicity, resulting in tachycardia, arrhythmia, convulsions, vomiting, coma, and possibly death. In particular, high caffeine consumption while pregnant can cause increased fetal catecholamine levels, which could lead to increased fetal heart rate and placental vasoconstriction and impair fetal oxygenation. Therefore, caffeine intoxication in pregnant women should be treated immediately. Herein, we present a 33-year-old pregnant woman who was treated in our department after ingesting 4000mg of caffeine in an attempt to commit suicide. We successfully treated our patient, and she delivered a healthy baby at 38 weeks.

## Introduction:

The widely-consumed psychoactive compound caffeine, 1,3,7-trimethylxanthine, is a natural xanthine alkaloid that stimulates the central nervous system (CNS). Caffeine functions as an adenosine receptor antagonist in moderate doses and as a phosphodiesterase inhibitor at high doses, and directly releases intracellular calcium at very high doses (1). In recent years, pure caffeine (powder or tablets) has become very easy to obtain on the Internet, increasing the risks of willfully or unwillfully ingesting, possibly deadly levels of caffeine (1-3).

Caffeine easily permeates the placenta. The fetus depends primarily on the mother’s caffeine clearance, which is decreased during pregnancy. Caffeine-induced increase of catecholamine concentrations impedes placental blood flow and hinders the transport of transplacental nutrients to the fetus (4). Therefore, clinicians need to pay attention to caffeine intoxication in pregnant women. Herein, we describe a pregnant woman who knowingly ingested a large number of caffeine tablets as part of a suicide attempt and the concentration of caffeine had reached a dangerous level, but her fetus was not effected by caffeine. We treated her with continuous hemodiafiltration (CHDF) because of her and her fetus’ caffeine concentration. Presentation of the detailed history, diagnosis process, and successful treatment of a case like this one may be a valuable contribution to the medical literature and may help emergency physicians formulate therapeutic strategies.

## Case Presentation:

A 33-year-old female who was 23 weeks pregnant was transferred to our emergency department complaining of nausea and vomiting. Her past medical history included hypothyroidism from 24- to 32 years-old, which was treated with thyroid hormone intake. No depression, psychological disorders, or previous overdoses were reported. The patient had become depressed around her 20^th^ week of pregnancy. She stated that she ingested 200 caffeine tablets containing approximately 4000 mg of caffeine obtained over-the-counter in a drugstore in a suicide attempt. Two hours after the self-poisoning, her mother found her vomiting and coughing and called emergency medical service. 

On arrival at the emergency department, the patient was oriented with a Glasgow Coma Scale score of 14 (E3V5M6). She complained of a severe headache and nausea. Vital signs included respiration rate of 16 breaths per minute, blood pressure of 111/60 mmHg, heart rate of 111 beats per minute, and fever of 36.5ºC. Her pupil size was bilaterally 4 mm with prompt light reflex. No abnormalities were noted during her systemic physical examination, and neurological tests for conditions like anisocoria, paralysis, or seizures were unremarkable. Her electrocardiogram presented supraventricular tachycardia without QT prolongation. Her blood gas analysis results were: pH: 7.431; PCO_2_: 36.2 mmHg; HCO_3_: 23.5 mmol/L; and lactate: 1.29 mmol/L. 

Her laboratory data was as follows: white blood cells, 10920/μL; hemoglobin, 11.6 g/dL; serum creatine kinase, 73 IU/L; sodium 136 mmol/L; potassium, 2.6 mmol/L; blood glucose 96 mg/dL; blood urea nitrogen 5.7 mg/dL; creatinine 0.66 mg/dL; total bilirubin 0.3 mg/dL; aspartate aminotransferase 19 IU/L; alanine aminotransferase 13 IU/L; and lactate dehydrogenase 170 IU/L. Although urine drug screening tests (Triage®, Biosite, San Diego, CA, USA) were negative, blood samples taken for toxicological examination on admission and revealed a high caffeine concentration (90.4 µg/mL). Fetal wellbeing was confirmed: heart rate 160 bpm, biparietal diameter 56.3 mm, abdominal circumference 17.1 cm, femur length 40.1 mm, and estimated fetal weight 564 grams.

As we diagnosed the patient with caffeine intoxication based on her interview, physical examination, and high blood caffeine concentration, gastric lavage and fluid resuscitation were performed. Since her blood pressure remained steadfastly low (systolic reading in the 80s) and she responded inadequately to catecholamine injection, after admission to the intensive care unit four hours after overdosing, CHDF with dialyzer of UT-1100 was initiated to remove caffeine from the circulation rapidly. The conditions of CHDF were as follows: blood flow rate of 80 mL/minute, filtration flow rate of 1200 mL/hr, dialysate flow rate of 800 mL/hr, and replacement flow rate of 400 mL/hr. 

After 12 hours of CHDF treatment, the patient’s clinical symptoms wholly recovered, with caffeine concentration decreasing to 54.3 µg/mL. The following day, her blood caffeine concentration was 32.5 µg/mL and returned to normal undetectable levels within three days. The patient recovered without sequelae and was sent to the local hospital’s obstetrics and gynecology ward on the seventh day after her overdose, with psychological follow-up, as she was taking paroxetine, etizolam, and flunitrazepam medications. At 38 weeks, she delivered a healthy infant weighing 2796 g, who had one- and five-minute Apgar scores of 8 and 9, respectively. Currently, both the infant and mother are doing well.　

## Discussion:

Caffeine consumption has continuously been increasing in Japan, especially among children and young adults, since caffeine or products containing caffeine are sold over-the-counter worldwide and can be purchased easily online. Caffeine’s growing accessibility can have serious health consequences. In general, deadly caffeine overdoses involve ingesting medications containing caffeine rather than beverages or foods containing caffeine (5) and have been related to blood concentrations exceeding 80 mg/L (6). Despite potential death from higher doses, caffeine is still rarely associated with attempted and successful suicides. However, we must be aware that the rate of caffeine-associated morbidity and mortality has recently been deemed a national crisis (2).

Caffeine is rapidly absorbed from the digestive tract with a bioavailability of almost 100% (7). Within 15 minutes, clinical effects are identifiable, and peak plasma levels are reached within 15–45 min after consumption. Caffeine overdose mainly affects renal, cardiovascular, and neurologic systems, which are mediated by the drug’s functions as an adenosine-receptor antagonist. Caffeine also functions as a phosphodiesterase inhibitor, which boosts the availability of cyclic adenosine monophosphate and intracellular calcium concentration, causes noradrenalin release, and sensitizes the dopamine receptors by augmenting sympathetic effects. Caffeine’s toxic consequences on the CNS include flushing, agitation, irritability, chills, impairment of consciousness, convulsions and rigidity, appetite loss, and weakness. At first, observations often reveal normotension, hypertension, or tachycardia followed by fever and hypotension. Early blood tests usually show few abnormalities, but can show metabolic acidosis, hypokalemia, and hyponatremia. Severe overdose often leads to dysrhythmia, including atrioventricular block, bradycardia, ventricular tachycardia, supraventricular tachycardia, and cardiac arrest due to ventricular fibrillation. 

We treated the caffeine toxin with CHDF. Although CHDF has not been previously used to manage patients with caffeine overdose, physicians may consider treating patients like ours, who are hypotensive, unstable, and unresponsive to conventional treatment, with CHDF, since it has little cardiovascular impact and is therefore safer for hypotensive patients (8).

Previous studies indicated that chronic caffeine intoxication causes abortion, intrauterine growth restriction, and attention-deficit hyperactivity disorder. However, the effect of acute caffeine intoxication on pregnancy and the fetus is not unclear. Caffeine is metabolized by the enzyme CYP1A2. Caffeine’s half-life is significantly longer in pregnant women due to lessened activity of CYP1A2 (9), and the fetus and newborn don't have the enzyme (10). Moreover, in pregnancy, caffeine reaches the fetus through the placenta (9). Therefore, high caffeine consumption while pregnant can increase fetal catecholamine levels, which can result in increased fetal heart rate and placental vasoconstriction, leading to impairment of fetal oxygenation (11). If the concentration of caffeine remains high in the fetus, the fetus may die due to caffeine intoxication, but fortunately, the fetus was doing well in the present case. Using treatments to rapidly decrease the concentration of caffeine, such as CHDF, can bring about good results for the mother and the fetus. Clinicians should be aware that caffeine intoxication may imitate acute pregnancy complications like seizures and eclampsia.

In conclusion, caffeine overdose during pregnancy can cause severe adverse effects in both the fetus or newborn and the mother. Fast diagnosis and management are needed to avert adverse outcomes.

**Consent**

Written informed consent was obtained from the patient’s legal guardian for publication of this case report and any accompanying images. A copy of the written consent is available for review by the Editor-in-Chief of this journal.
